# Predicting recalcitrant hyperinflammatory disease course in children with Kawasaki disease and MIS-C

**DOI:** 10.1186/s12969-026-01206-7

**Published:** 2026-03-24

**Authors:** Özlem Satirer, Fehime Kara Eroglu, Johannes Nordmeyer, Matthias Kumpf, Felix Neunhoeffer, Vanya Icheva, Christiane Reiser, Oana Buzoianu, Susanne M. Benseler, Jasmin B. Kuemmerle-Deschner

**Affiliations:** 1 Paediatric Rheumatology, Department of Paediatrics and Autoinflammation Reference Center Tuebingen (arcT), University Hospital Tuebingen, Tuebingen, Germany; 2 Department of Pediatric Cardiology, University Children’s Hospital, University of Tübingen, Tübingen, Germany; 3 Department of Paediatrics, Cumming School of Medicine, University of Calgary, Calgary, AB Canada; 4 Children’s Health Ireland, University College Dublin & Trinity College Dublin, Dublin, Ireland; 5 Department of Pediatrics, Landeskrankenhaus Bregenz, Carl-Pedenz-Str. 2, Bregenz, 6900 Austria; 6 Division of Pediatric Rheumatology, Department of Pediatrics, University Children’s Hospital Tuebingen, Hoppe-Seyler-Strasse 1, 72076 Tuebingen, Germany

**Keywords:** IL-2R, NT-proBNP, SAA, Hyperinflammation, Biomarkers, disease course

## Abstract

**Background:**

Hyperinflammation ranges from monophasic to rapidly progressive, life-threatening courses. Early biomarkers to identify high-risk children are needed.

**Methods:**

This single-center cohort included consecutive children with hyperinflammation between 01/2021 and 01/2024. Demographic, clinical, laboratory, cardiac imaging, and treatment data were analysed.

**Results:**

Of 80 patients, 56 (70%) had a recalcitrant course. Fever was universal. Rash, mucosal involvement, and conjunctivitis were more common in the monophasic group, while abdominal pain, neurological signs, pleural effusion, lymphopenia, thrombocytopenia, hypoalbuminemia, and elevated ferritin characterised the recalcitrant group (*p* < 0.01). Myocarditis and reduced ejection fraction occurred only in the recalcitrant cohort; coronary artery changes were more frequent in monophasic cases (21% vs. 5%). All survived. All received IVIG; steroids and anakinra were used only in recalcitrant cases, who also had longer hospital stays, ICU admissions (21% vs. 0%, *p* < 0.01), and required inotropes/ventilation. Baseline SAA, IL-2R, and NT-proBNP were significantly higher in the recalcitrant cohort. In ROC analyses, NT-proBNP showed the highest diagnostic accuracy (AUC 0.912), followed by IL-2R (AUC 0.873) and SAA (AUC 0.793) (all *p* < 0.001).

**Conclusions:**

NT-proBNP, IL-2R, and SAA were strongly associated with a recalcitrant hyperinflammatory course and may aid early prognostication and monitoring.

## Background

Post-infectious hyperinflammation encompasses a broad clinical spectrum, ranging from mild inflammation to life-threatening complications such as cardiogenic shock, macrophage activation syndrome (MAS), and multi-organ dysfunction requiring intensive care [1–4]. Within this spectrum, Kawasaki disease and multisystem inflammatory syndrome in children (MIS-C) have been identified as two key entities [5]. Kawasaki disease, including its incomplete form, is a systemic vasculitis triggered by various infectious agents, typically self-limiting or monophasic, and often responsive to intravenous immunoglobulin (IVIG) therapy. However, in some cases, it may lead to complications such as coronary artery aneurysms and shock [6]. MIS-C is a hyperinflammatory syndrome following SARS-CoV-2 infection, characterized by progressive systemic inflammation, multi-organ involvement, and a higher incidence of shock [6]. Although these conditions have traditionally been considered distinct clinical entities, recent genomic and immunological studies suggest that they may represent different manifestations of a shared immune response spectrum [5–7]. Artificial intelligence-assisted analyses of gene expression signatures indicate that both conditions involve a similar host immune response, with differences in disease severity rather than fundamentally distinct pathophysiological mechanisms [7]. Transcriptomic profiling has demonstrated overlapping activation of innate and adaptive immune pathways in KD and MIS-C, including monocyte/macrophage activation, expansion of activated T cell subsets, and convergence on an IL-15/IL-15RA–centric cytokine network. These shared upstream inflammatory signatures support the concept of a common hyperinflammatory immune phenotype, whereas differences in clinical presentation—such as the higher frequency of myocardial dysfunction and shock in MIS-C—appear to reflect variation in the magnitude and systemic distribution of immune activation rather than entirely distinct immunopathogenic processes [5, 7].

A significant challenge in management of hyperinflammatory syndromes is the limited ability to predict which patients will progress to severe, recalcitrant disease course. Clinical criteria alone are inadequate for distinguishing individuals who will experience spontaneous resolution from those at risk of life-threatening complications [5]. Some patients present with mild fever and inflammation that resolve without intervention, while others deteriorate quickly, requiring intensive care. Given this uncertainty, there is a critical need for reliable prognostic markers to identify high-risk patients early and guide timely therapeutic escalation. Despite numerous efforts to establish diagnostic and prognostic markers, no definitive biomarker has yet been identified. Therefore, ongoing research aims to refine risk stratification and improve early detection of high-risk patients within post-infectious hyperinflammatory spectrum [8].

The aims of this study were:

1. to describe a longitudinal cohort of consecutive paediatric patients with Kawasaki disease and multisystem inflammatory syndrome in children (MIS-C) presenting with hyperinflammation,
2. to compare presenting clinical and laboratory parameters between children with IVIG-responsive (monophasic) disease course and those with IVIG-refractory (recalcitrant) disease course, and.
3. to identify biomarkers predicting disease course and to analyse their trajectories in response to therapy.

## Methods

This single-center longitudinal study included consecutive paediatric patients with hyperinflammation between January 2021 and January 2024. Patients were included, if they presented with clinical and laboratory features of hyperinflammation encompassing the broad spectrum of Kawasaki disease (KD) and multisystem inflammatory syndrome in children (MIS-C). Children were excluded if they were found to have microbiological evidence of sepsis or other active infections, or mimics such as appendicitis. Data were prospectively recorded in institutional Arthritis and Rheumatism Database and Information System (ARDIS). Ethical approval was obtained from the Ethics Committee of the Medical Faculty at Eberhard Karls University and the University Hospital Tuebingen (Project No: 782/2023BO2). The study was conducted in accordance with the Declaration of Helsinki. Written informed consent was obtained from the patient(s) and/or their legal guardians for participation in this study.

### Data

**Clinical characteristics** recorded included age at diagnosis, sex, country of family origin, admission diagnosis, duration of fever on admission, presence of rash, mucous membrane involvement, conjunctivitis, hand and/or foot erythema/edema, cervical lymphadenopathy, abdominal pain, pleuritic chest pain and neurological involvement. Length of hospital stay and need for intensive care unit (ICU) admission, inotropic support, and/or mechanical ventilation were recorded. Therapies including type, dose and duration were captured. **Laboratory data** included complete blood count (CBC) and differential, including haemoglobin, total white blood cell (WBC), platelet, absolute lymphocyte count, and biochemical parameters including sodium, aspartate aminotransferase (AST), alanine aminotransferase (ALT), creatinine, albumin, and troponin levels. Urinalysis was performed to assess for pyuria, defined as ≥ 10 white blood cells per high-power field (WBC/hpf). **Traditional inflammatory markers** included erythrocyte sedimentation rate (ESR), C-reactive protein (CRP) and ferritin. **Potential inflammatory markers** captured were interleukin-2 receptor (IL-2R), interleukin-6 (IL-6) plasma levels, N-terminal pro b-type Natriuretic Peptide (NT-proBNP), and serum amyloid A (SAA). **Microbiological evaluations** included SARS-CoV-2 PCR testing and COVID-19 antibody detection. **Cardiac imaging features** on echocardiogram (ECHO) included left ventricular ejection fraction (LVEF), evidence of mitral and/or tricuspid regurgitation, and coronary artery lesions (CAL). These were classified as dilation for z-scores between 2 and < 2.5, and as aneurysm for z-scores ≥ 2.5 [9]. Aneurysms were further categorized based on z-scores and absolute dimensions as follows: small aneurysm (z-score ≥ 2.5 to < 5), medium aneurysm (z-score ≥ 5 to < 10 and absolute dimension < 8 mm), and large or giant aneurysm (z-score ≥ 10 or absolute dimension ≥ 8 mm) [9]. Electrocardiogram (ECG) was also performed. Study visits were defined as follows: (1) **baseline visit** corresponded to the hospital admission before treatment initiation; (2) **therapy escalation visit** occurred when conventional treatment (IVIG) failed, prompting the introduction of high-dose corticosteroids and/or biologic therapy; (3) **discharge visit** was defined as the final assessment before hospital discharge; and (4) the **last visit** referred to the final follow-up within the study period.

### Definitions

#### Hyperinflammation syndromes

In this study, Kawasaki disease (complete and incomplete forms) and multisystem inflammatory syndrome in children (MIS-C) were considered the primary diagnoses of the study population. Hyperinflammation was defined as a shared clinical inflammatory phenotype, characterized by systemic immune activation and variable organ involvement, observed in these conditions. **MIS-C** was defined according to WHO 2020 criteria, requiring fever > 3 days and at least two of the following: rash or mucocutaneous inflammation, hypotension/shock, cardiac involvement (myocardial dysfunction, pericarditis, valvulitis, or coronary abnormalities), coagulopathy (PT, PTT, or elevated d-dimers), or acute gastrointestinal symptoms. Diagnosis also required elevated inflammatory markers (ESR, CRP, or procalcitonin), absence of an alternative microbial cause, and evidence of COVID-19 infection or exposure [10]. **Complete and incomplete KD** were defined based on American Heart Association (AHA) criteria. Complete KD required fever ≥ 5 days and at least four of five clinical features: bilateral conjunctival injection, oral mucosal changes, extremity changes, rash, and/or cervical lymphadenopathy > 1.5 cm [11]. Incomplete KD was diagnosed in children with prolonged fever and fewer than four criteria but with supporting laboratory and/or echocardiographic findings [12].

**The monophasic course** of hyperinflammation was defined as IVIG-responsive and not requiring therapy escalation such as high-dose corticosteroids, and/or biologic agents. **The recalcitrant course** was defined as progression despite IVIG and requirement for therapy escalation.

### Outcome

The primary outcome was recalcitrant disease course defined as progression of hyperinflammation despite IVIG and requirement for therapy escalation. Secondary outcomes included (1) overall survival, (2) length of hospital stay, (3) duration of fever, and (4) requirement for ICU admission and/or need for inotropic support and/or need for mechanical ventilation.

### Statistical analysis

Descriptive statistics summarized patient characteristics. Categorical variables were reported as counts and percentages; continuous variables as means (SD) or medians (range), based on distribution. Normality was assessed using Shapiro-Wilk or Kolmogorov-Smirnov tests. Group comparisons used independent t-tests or Mann-Whitney U tests. For longitudinal analysis, paired t-tests and repeated measures ANOVA were applied. Receiver operating characteristic (ROC) analysis assessed model accuracy via area under the curve (AUC), sensitivity, specificity, and Youden’s index. To reduce the risk of false-positive findings in the context of multiple comparisons, a more stringent significance threshold (*p* < 0.01) was applied for the selection of candidate variables entering ROC and multivariable analyses. Analyses were performed using SPSS v28.0.1.1 and STATsoftware.

## Results

The study included a total of 80 children, comprising 51 males (64%) and 29 females (36%). Median age of the cohort was 5 years (1–16). COVID-19 antibodies were detected in 69 patients (86%) overall. A total of 56 children (70%) experienced a recalcitrant disease course, while 24 (30%) had a monophasic course of hyperinflammation. Among the recalcitrant cohort, 44 (79%) had a physician diagnosis of MIS-C at admission; 12 (21%) were classified as Kawasaki/MIS-C overlap. All patients in the monophasic cohort were diagnosed with Kawasaki disease. All children survived.

Patients with a recalcitrant course were generally older and more frequently had detectable COVID-19 antibodies. Sex and country of origin were similar across both groups. The mean follow-up was 20 months (3–36) in the monophasic cohort and nine (2–36) in the recalcitrant cohort (Table 1).

**Table 1:** Patient- and treatment-related characteristics of children with distinct disease courses of systemic hyperinflammation

Characteristics	Total cohort (*n* = 80)	Monophasic disease course (*n* = 24)	Recalcitrant disease course (*n* = 56)
Age at diagnosis in years, median (range)	5 (1–16)	2 (1–10)	6 (1–16)*
Male sex, n (%)	51 (64%)	15 (63%)	36 (64%)
Origin, German, n (%)	77 (96%)	24 (100%)	53 (95%)
COVID-19 antibody positivity, n (%)	69 (86%)	13 (54%)	56 (100%)*
**Course in hospital**			
ICU admission, n (%)	12 (15%)	0	12 (21%)*
Inotropic support, n (%)	7 (9%)	0	7 (13%)*
Mechanical ventilation, n (%)	6 (7%)	0	6 (11%)*
Overall length of hospital stays in days, median (range)	7 (2–56)	5 (2–20)	8 (3–56)*
**Clinical features**			
Fever, n (%)	80 (100%)	24 (100%)	56 (100%)
Duration of fever in days, median (range)	10 (3–40)	9 (5–15)	10 (3–40)
Rash, n (%)	50 (63%)	22 (93%)	28 (50%)*
Mucous membrane involvement, n (%)	47 (59%)	18 (75%)	29 (52%)*
Non-purulent conjunctivitis, n (%)	44 (55%)	21 (88%)	23 (41%)*
Hand and foot erythema/edema, n (%)	32 (40%)	12 (50%)	20 (36%)*
Cervical lymphadenopathy, n (%)	46 (58%)	16 (66%)	30 (54%)
Abdominal pain, n (%)	18 (23%)	4 (17%)	14 (25%)
Pleural effusion, n (%)	13 (16%)	0	13 (23%)*
Neurological involvement, n (%)	6 (7%)	0	6 (11%)*
**Cardiac imaging features**			
Myocarditis features, n (%)	24 (30%)	0	24 (43%)*
Reduced left ventricular ejection fraction, n (%)	12 (15%)	0	12 (21%)*
Mitral regurgitation, n (%)	7 (8%)	0	7 (12%)*
Tricuspid regurgitation, n (%)	3 (3%)	0	3 (5%)*
Coronary artery lesions, n (%) • Dilatation, n (%) • Aneurysm, n (%)	12 (15%) 4 (5%) 8 (10%)	7 (29%) 2 (8%) 5 (21%)	5 (8%)* 2 (3%) 3 (5%)
**Treatment**			
Intravenous immunoglobulin (IVIG), n (%)	80 (100%)	24 (100%)	56 (100%)
Acetylsalicylic acid, n (%)	51 (64%)	20 (83%)	31 (55%)*
High-dose corticosteroids, n (%)	36 (45%)	0	36 (64%)*
Anakinra, n (%)	19 (24%)	0	19 (34%)*

Legend: * Statistically significant difference (p < 0.01). Group comparisons were performed using independent t-tests for normally distributed data and the Mann-Whitney U test for non-normally distributed data

### Clinical and laboratory characteristics

Fever was present in all children, with a similar median duration in both cohorts, 9 versus 10 days, respectively. The monophasic cohort more commonly exhibited rash (93% vs. 50%, *p* < 0.001), mucous membrane involvement (75% vs. 52%, *p* = 0.049), conjunctivitis (88% vs. 41%, *p* < 0.01), hand and/or foot erythema/edema (50% vs. 36%, *p* < 0.01) and cervical lymphadenopathy (66% vs. 54%, *p* = 0.04). In contrast, the recalcitrant cohort had more frequently evidence of abdominal pain (25% vs. 17%, *p* = 0.037), neurological involvement (11% vs. 0, *p* < 0.001) and pleural effusions (23% vs. 0, *p* < 0.01).

All children had evidence of hyperinflammation. Haemoglobin levels were comparable between the cohorts (*p* = 0.56). Similarly, total WBC count did not differ significantly (10.2 vs. 11.6 × 10³/L, *p* = 0.607). In contrast, platelet counts were significantly lower in the recalcitrant cohort (298 vs. 525 × 10³/L, *p* < 0.01), as was the absolute lymphocyte count (2024 vs. 3504/µL, *p* < 0.01). Troponin levels showed a trend toward higher values in the recalcitrant cohort not reaching statistical significance (199.9 vs. 44.4, *p* = 0.09). There were significant differences seen for creatinine, sodium and albumin levels between cohorts, while AST and ALT levels were similar. Pyuria was more frequent in the monophasic disease course (52% vs. 13%, *p* < 0.01) (see Table 2).

### Traditional and potential inflammatory markers

**Fig. 1:**
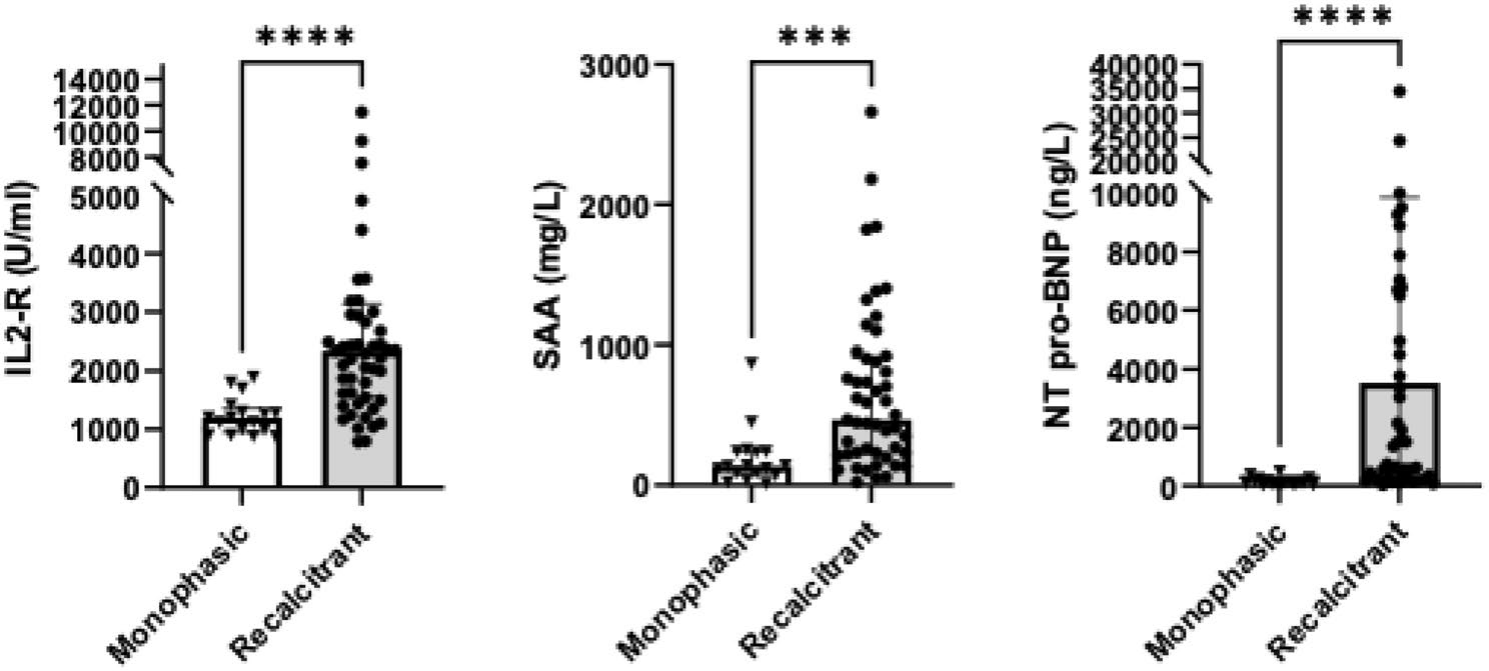
Performance of potential biomarkers in differentiating monophasic from recalcitrant disease course in children. Legend: IL-2R (2868 vs. 1225 U/mL, p < 0.01), NT-proBNP (3532 vs. 147 ng/L, p < 0.01), and SAA levels (660 vs. 191.5 mg/L, p < 0.01) were significantly higher in recalcitrant disease course

Traditional inflammatory markers including ESR and CRP levels did not differ between cohorts. However, ferritin levels were significantly higher in the recalcitrant cohort (40.3 vs. 11.7 µg/L, *p* = 0.021). Potential biomarkers distinguished monophasic from recalcitrant disease courses: At baseline, IL-2R, NT-proBNP, and SAA levels were found to be significantly higher in the recalcitrant cohort (2868 vs. 1225 U/mL, *p* < 0.01; 3532 vs. 147 ng/L, *p* < 0.01; 660 vs. 191.5 mg/L, *p* < 0.01, respectively), while IL-6 levels did not differ (257 vs. 81.6 ng/L, *p* = 0.89) (Table 2) (Fig. 1).

### Cardiac evaluation

Overall, CALs were observed in 15% of patients, including 5% having dilation and 10% aneurysms on ECHO. Myocarditis was detected in 30% overall. It was significantly more prevalent among patients with a recalcitrant disease course compared to those with a monophasic course (43% vs. 0%, *p* < 0.01). Similarly, reduced left ventricular ejection fraction (21% vs. 0%, *p* < 0.01), mitral regurgitation (12% vs. 0%, *p* < 0.01), and tricuspid regurgitation (5% vs. 0%, *p* < 0.01) were solely observed in the recalcitrant disease course cohort. In contrast, coronary artery involvement was more commonly detected in the monophasic disease course cohort (29% vs. 8%, *p* < 0.01), with higher rates of coronary artery dilatation (8% vs. 3%, *p* = 0.04) and aneurysms (21% vs. 5%, *p* = 0.02) (Table 1).

In addition, elevated NT-proBNP was more frequently observed in patients with a recalcitrant disease course. Among the 56 recalcitrant patients, 38 (68%) showed elevated NT-proBNP, whereas myocarditis was present in 24 (43%) and reduced LVEF in 12 (21%). In contrast, among the 24 monophasic patients, 12 (50%) had elevated NT-proBNP, while none showed myocarditis or reduced LVEF.

### Course in hospital and treatment

Overall, the median hospital stay was seven days (2–56) with 12 children (15%) requiring ICU admission. Patients with a recalcitrant disease course stayed significantly longer (8 vs. 5 days, *p* = 0.04). ICU admission (21% vs. 0%, *p* < 0.01), inotrope use (13% vs. 0%), and mechanical ventilation (6% vs. 0%, *p* < 0.01) were solely required in children with recalcitrant disease course.

All patients received IVIG as first-line therapy. Aspirin use was lower in patients with a recalcitrant disease course (55% vs. 83%, *p* = 0.034). High-dose steroids were administered to 64% of the recalcitrant cohort, whereas none in the monophasic cohort received steroids (*p* < 0.01). Anakinra was exclusively required in patients with a recalcitrant disease course (34% vs. 0%, *p* < 0.01) (Table 1). At the last visit, nine children (38%) with monophasic and five (9%) with recalcitrant disease course remained on aspirin due to coronary artery involvement. One patient in the recalcitrant cohort continued anakinra for mild persistent disease activity.

### Performance of potential biomarkers in predicting disease course

**Fig. 2:**
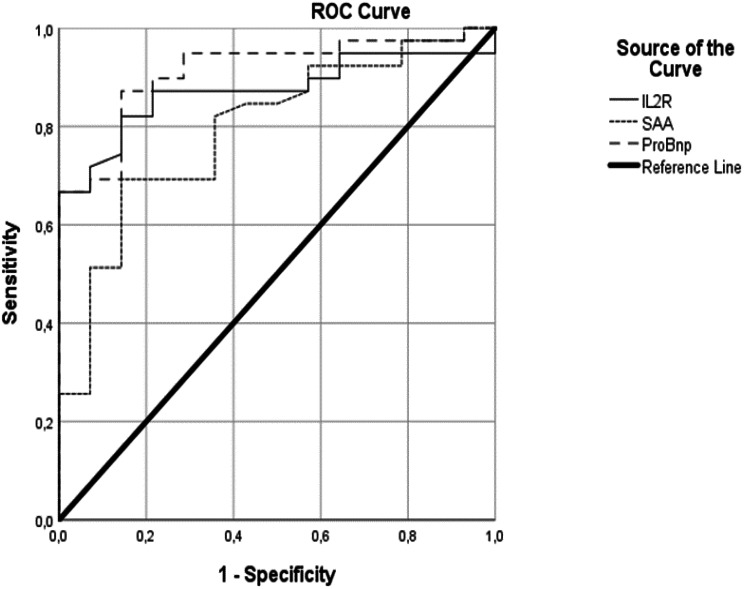
Predictive utility of IL-2R, SAA, and NT-proBNP in disease course differentiation: A ROC curve-based model analysis. Legend: NT-ProBNP demonstrated the highest diagnostic accuracy (AUC: 0.912, 95% CI: 0.832–0.992, p < 0.001), followed by IL-2R (AUC: 0.873, 95% CI: 0.778–0.967, p < 0.001) and SAA (AUC: 0.793, 95% CI: 0.661–0.925, p = 0.001)

Biomarkers found to be significantly associated with recalcitrant disease course included NT-proBNP, IL-2R, and SAA (Table 2). In ROC curve analyses NT-proBNP demonstrated the highest diagnostic accuracy (AUC: 0.912, 95% CI: 0.832–0.992, *p* < 0.001), followed by IL-2R (AUC: 0.873, 95% CI: 0.778–0.967, *p* < 0.001) and SAA (AUC: 0.793, 95% CI: 0.661–0.925, *p* = 0.001) (Fig. 2). Optimal cut-off values based on ROC analysis for SAA was 168.5 mg/L, yielding a sensitivity of 0.821, a specificity of 0.643, and a Youden’s Index of 0.464. For IL-2R, a cut-off of 1429 U/mL provided a sensitivity of 0.82 and a specificity of 0.85, resulting in a Youden’s Index of 0.67. NT-proBNP exhibited the highest discriminatory power with an optimal cut-off of 171 ng/L, achieving a sensitivity of 0.87, a specificity of 0.85, and a Youden’s Index of 0.72. (Fig. 2).

### Biomarkers trajectories in response to therapy

**Fig. 3:**
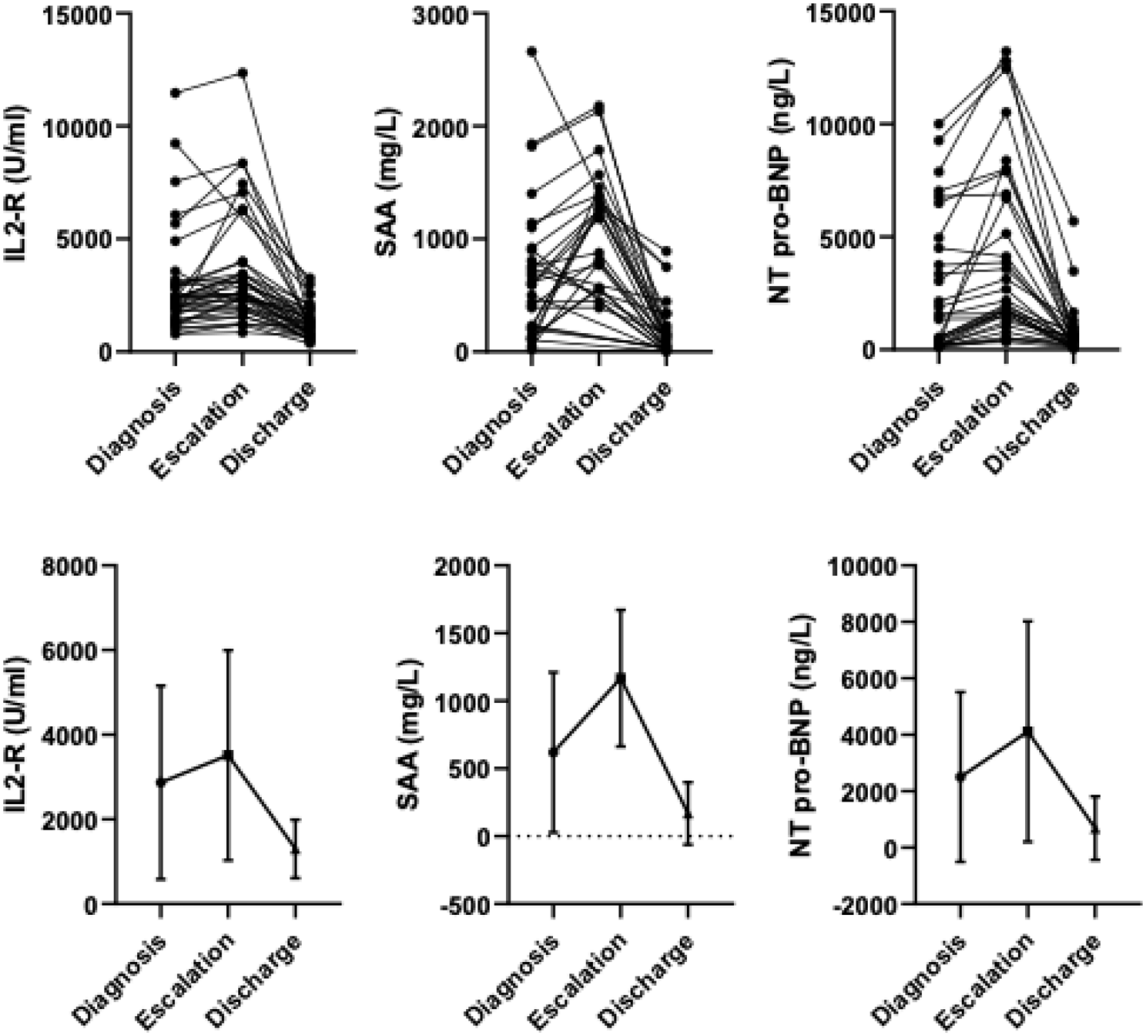
Trajectories of biomarkers in response to therapy escalation in children with recalcitrant hyperinflammation. Legend: Trajectories of SAA, IL-2R, and NT-proBNP levels in 41 patients with a recalcitrant disease course requiring therapy escalation. A significant time effect was observed (p < 0.001), with linear and quadratic trends. SAA, IL-2R, and NT-proBNP levels increased at therapy escalation and declined before discharge. Abbreviations: SAA = Serum Amyloid A, IL-2R = Interleukin-2 Receptor, NT-proBNP = N-terminal pro b-type Natriuretic Peptide

To assess biomarker responsiveness, patients with a recalcitrant disease course requiring therapy escalation were analyzed (*n* = 41). Among these, 31 patients received high-dose corticosteroids only, 4 patients received Anakinra only, and 6 patients received both agents. Subgroup analyses stratified by specific therapy did not reveal statistically significant differences between treatment groups; nonetheless, all three biomarkers demonstrated responsiveness to therapy escalation. Overall, SAA levels were 639 mg/L (range: 21–2660) at baseline, increased to 1120.0 mg/L (480–1780) at time of therapy escalation, and declined to 159.0 mg/L (1–890) prior to discharge. IL-2R levels were 2870 U/mL (783–11,464) at baseline, rose to 3515 U/mL (850–12,356) at time of escalation, and decreased to 1301 U/mL (363–3259) before discharge. Similarly, NT-proBNP concentrations were 4024 ng/L (77–34,514) at baseline, increased to 5596 ng/L (340–35,678) during escalation, and declined to 667 ng/L (43–5698) prior to discharge (Fig. 3). A significant effect of time on biomarker levels was observed for SAA, IL-2R, and NT-proBNP (Pillai’s trace = 0.844, 0.525, 0.603; all *p* < 0.001). Mauchly’s test indicated violations of sphericity (*p* = 0.042, < 0.001, and 0.008, respectively), and Greenhouse-Geisser corrections were applied where appropriate. Repeated measures ANOVA demonstrated a robust effect of time on biomarker levels (F = 37.258, 27.155, 21.983; all *p* < 0.001), with statistically significant linear trends (F = 10.956, 18.074, 11.523; *p* = 0.003, < 0.001, 0.002) and quadratic trends (F = 108.075, 39.418, 27.346; all *p* < 0.001) across all three biomarkers underscoring the temporal dynamics of biomarker changes in response to therapeutic interventions.

## Discussion

This comprehensive study has identified sensitive and responsive biomarkers of hyperinflammation that were able to predict disease course in children. While the systematic evaluation of clinical, laboratory, and imaging data enables the recognition of hyperinflammation, the disease course and therefore the selection and escalation of treatments remains challenging. Addressing the clinical ambiguity at diagnosis of hyperinflammation the study explored the utility of Potential biomarkers in early prediction of recalcitrant disease course. NT-proBNP, IL-2R and SAA emerged as the most promising biomarkers. The application of these biomarkers in clinical practice could enable the early identification of high-risk patients, thereby facilitating timely and targeted treatment decisions aimed at effectively controlling hyperinflammation.

Since the COVID-19 pandemic, hyperinflammatory diseases are increasingly recognized. In children, these conditions span a wide clinical spectrum, ranging from mild to life-threatening presentations. Although distinct clinical and laboratory features have been described, overlapping phenotypes and the limited prognostic utility of conventional clinical and laboratory markers have posed significant challenges in predicting disease course. In this study, monophasic disease course was present in younger children, while recalcitrant disease course was more common in adolescents, consistent with existing literature [13, 14]. One hypothesis attributed this age disparity to induced immune memory, in which an increase in central and effector memory CD4 + T cells and a reduction in naive CD4 + T cells may contribute [15]. Males were more frequently affected, consistent with prior studies reporting a higher KD prevalence in boys. In MIS-C, the male-to-female ratio is 1:1 between 0 and 4 years of age but rises to 2:1 in patients aged 18–20 years [13]. Monophasic course was associated with typical mucosal and skin involvement, including maculopapular rash, erythema, edema, and non-exudative conjunctivitis. In contrast, recalcitrant course was characterized by more extensive organ involvement, longer hospitalizations, and a greater need for intensive care, inotropic support, and mechanical ventilation [13, 14]. A meta-analysis by Tong et al., involving 2,928 patients, confirmed a higher incidence of shock and systemic involvement in progressive/multisystemic hyperinflammatory diseases [16]. Cardiac abnormalities, including myocarditis, valvular dysfunction, and decreased ejection fraction, were more frequently observed in patients with recalcitrant disease. In contrast, coronary artery abnormalities were predominantly seen in monophasic disease course, aligning with existing literature [13, 14]. Recalcitrant disease was also associated with higher levels of creatinine, ALT, AST, CRP, and ferritin, alongside lower lymphocyte and platelet counts and reduced albumin [16]. Although ferritin differed between groups at *p* < 0.05, a more stringent pre-specified significance threshold (*p* < 0.01) was applied for the selection of candidate predictors entering ROC and multivariable analyses in order to reduce false-positive findings in the context of multiple comparisons. Therefore, ferritin was not prioritized for predictive modelling. In addition, patients with overt macrophage activation syndrome were not included in this cohort, which may further explain why ferritin did not emerge as a decisive prognostic biomarker in this setting. Overall, the prognostic performance of the investigated biomarkers remained limited. This underscores the need for further research to address the clinical uncertainties in hyperinflammation at diagnosis and explore the potential of Potential biomarkers for reliable early prediction of disease course.

The study identified early predictors of disease course and markers of treatment responsiveness – these were NOT predictors of CAL. The absence of an association with CAL may suggest that systemic inflammation–related biomarkers capture disease severity but not the vessel-specific mechanisms underlying coronary artery pathology. This supports the concept of partially distinct systemic hyperinflammatory and vasculitis-dominant phenotypes within the KD/MIS-C spectrum. Potential inflammatory markers, particularly NT-proBNP, IL-2R, and SAA, demonstrated excellent utility in early prognostic stratification of systemic hyperinflammation in children. Among them, NT-proBNP exhibited the highest diagnostic accuracy, IL-2R and SAA also contributed important information in distinguishing disease courses. NT-proBNP, a cardiac biomarker released in response to myocardial stress, is traditionally used in the assessment of heart failure Consistent with prior reports, elevated NT-proBNP and cardiac troponin levels have been described as distinguishing features between MIS-C and Kawasaki disease [16, 17]. In our cohort, elevated NT-proBNP was predominantly observed in patients with recalcitrant disease. Myocarditis and reduced LVEF occurred exclusively in this group; however, many patients with elevated NT-proBNP showed no overt cardiac dysfunction. Among recalcitrant patients, NT-proBNP elevation was more frequent than myocarditis or reduced LVEF, whereas in monophasic patients, NT-proBNP elevation occurred without any cardiac involvement. The discordance between NT-proBNP elevation and objective measures of cardiac dysfunction suggests that NT-proBNP elevation in this cohort may not solely reflect myocardial impairment. Taken together, these findings suggest that NT-proBNP may reflect systemic hyperinflammation rather than cardiac dysfunction alone [17]. Similarly, IL-2R – a marker of T-cell activation classically associated with hemophagocytic lymphohistiocytosis and macrophage activation syndrome – was significantly increased in patients with a progressive disease course. This increase was accompanied by lymphopenia and reduced NK and T-cell counts, reflecting underlying immune dysregulation. Previous studies have also documented elevated IL-2R in severe SARS-CoV-2 infections, further supporting its role in hyperinflammation [18]. Importantly, none of these biomarkers showed a consistent association with coronary artery involvement. The integration of these biomarkers into clinical practice may facilitate the early identification of high-risk patients, thereby supporting timely, targeted therapeutic interventions designed to effectively manage hyperinflammation.

This study has several limitations. It is a single-center cohort study, which may limit its generalizability, although the tertiary care center serves both the city and the surrounding region. The study had a limited sample size. However, the use of an electronic patient record system ensured data consistency, maintaining high quality despite the inherent limitations of retrospective studies. Being an observational study, the absence of standardized diagnosis and treatment protocols led to variability in therapies, though standardized outcome assessments, including echocardiography and biomarkers, were employed across all patients. In addition, established pathophysiological differences between Kawasaki disease and MIS-C—such as the higher prevalence of myocardial dysfunction and shock in MIS-C and the vasculitis-dominant phenotype in KD—may have contributed to biological and clinical heterogeneity within the combined analytical model. While our spectrum-based framework assumes shared upstream immune activation, disease-specific downstream manifestations could have influenced biomarker profiles and severity metrics.

## Conclusion

Early identification of high-risk patients with a recalcitrant disease course is crucial for making informed care decisions, including guiding timely therapeutic escalation. NT-pro-BNP, IL-2R, and SAA have emerged as promising Potential biomarkers for early prognostic differentiation and longitudinal monitoring. Multicenter prospective studies are needed to validate these findings and establish international consensus.

**Table 2:** Comparison of presenting laboratory findings of children with a monophasic and recalcitrant disease course of hyperinflammation

Parameter	Monophasic disease course (*n* = 24)	Recalcitrant disease course (*n* = 56)	*p*-value
**Complete blood count and differential**			
Hemoglobin, g/dL	10.8 (8–12.5)	10.8 (6.7–18.8)	0.56
Total WBC count, 10³/µL	10.2 (0.5–23.8)	11.6 (2.5–37.4)	0.607
Platelet count, 10³/µL	525 (210–1790)	298 (62–826)	< 0.01
Absolute lymphocyte count, /µL	3504 (1000–10000)	2024 (100–8700)	< 0.01
**Biochemistry/organ function parameters**			
Sodium, mEq/L	134 (129–144)	132 (126–142)	0.045
AST, U/L	53 (18–405)	119 (11–2270)	0.92
ALT, U/L	74 (15–608)	89 (8–2019)	0.42
Creatinine, mg/dL	0.2 (0.1–0.8)	0.47 (0.1–3.7)	0.037
Albumin, g/dL	3.3 (2.2–4.6)	2.9 (1.7–4.1)	0.01
Pyuria (≥ 10 WBC/hpf), n (%)	15 (52)	6 (13)	< 0.01
Troponin, pg/mL	44 (0–455)	199 (0–3069)	0.09
**Traditional inflammatory markers**			
ESR, mm/h	48 (2–90)	51 (2–120)	0.83
CRP, mg/dL	8.9 (0.01–15.3)	12 (0.57–25.2)	0.072
Ferritin, µg/L	11.7 (2–25)	40.3 (1.7–333)	0.021
**Potential inflammatory markers**			
IL-2R, U/mL	1225 (880–1890), *n* = 17	2868 (783–11464), *n* = 52	< 0.01
IL-6, ng/L	81.6 (11–173.2), *n* = 8	257 (3–3847), *n* = 36	0.89
NT-proBNP, ng/L	147 (17–495), *n* = 18	3532 (36–34514), *n* = 48	< 0.01
Serum Amyloid A, mg/L	191.5 (12–868.5), *n* = 17	660 (21–2660), *n* = 49	< 0.01

Legend: Normal ranges were as follows: NT-proBNP, 0–300 ng/L; IL-6, 0–4 ng/L; platelet count, 154000–386000/µL; creatinine, 0.5–0.8 mg/dL; absolute lymphocyte count, 1.1–2.8 × 10³/µL; and total white blood cell count, 4800–10100/µL. The upper reference limits for AST and ALT were 39 U/L and 29 U/L, respectively. Albumin, 3–5 g/dL; ferritin, 1–20 µg/L; serum amyloid A, ≤10 mg/L; IL-2R, 158–613 U/mL; sodium, 135–145 mEq/L; and troponin, 24–30 pg/mL. Group comparisons were performed using the independent samples t-test for normally distributed variables and the Mann–Whitney U test for non-normally distributed variables

## Data Availability

Data are available upon reasonable request.

## Abbreviations

AUC: Area under the curve
ALT: Alanine aminotransferase
AST: Aspartate aminotransferase
CBC: Complete blood count
CAL: Coronary artery lesion
CRP: C–reactive Protein
COVID: 19–coronavirus disease 2019
ECHO: Echocardiogram
ECG: Electrocardiogram
EF / LVEF: Left ventricular ejection fraction
ESR: Erythrocyte sedimentation rate
ICU: Intensive care unit
IL: 2R–Interleukin–2 receptor
IL: 6–Interleukin–6
IVIG: Intravenous immunoglobulin
KD: Kawasaki disease
MAS: Macrophage activation syndrome
MIS: C–Multisystem inflammatory syndrome in children
NT: proBNP–N–terminal pro–B–type natriuretic peptide
PCR: Polymerase chain reaction
PT: Prothrombin time
PTT: Partial thromboplastin time
ROC: Receiver operating characteristic
SAA: Serum amyloid A
SARS: CoV–2–severe acute respiratory syndrome coronavirus 2
SD: Standard deviation
WBC: White blood cell
WHO: World Health Organization
